# Construction of refined staging classification systems integrating FIGO/T‐categories and corpus uterine invasion for non‐metastatic cervical cancer

**DOI:** 10.1002/cam4.6179

**Published:** 2023-06-16

**Authors:** Xiao‐Dan Huang, Kai Chen, Liu Shi, Ying‐Shan Luo, Yi Ou‐Yang, Jun‐Yun Li, Lan‐Qing Huo, Lin Huang, Fo‐Ping Chen, Xin‐Ping Cao

**Affiliations:** ^1^ Department of Radiation Oncology; State Key Laboratory of Oncology in South China; Collaborative Innovation Center for Cancer Medicine; Guangdong Key Laboratory of Nasopharyngeal Carcinoma Diagnosis and Therapy Sun Yat‐Sen University Cancer Center Guangzhou China; ^2^ Department of Radiation Oncology Guangzhou Concord Cancer Center Guangzhou China

**Keywords:** cervical cancer, corpus uterine invasion, FIGO stage, recursive partitioning analysis, TNM stage

## Abstract

**Background:**

To investigate the prognostic value of corpus uterine invasion (CUI) in cervical cancer (CC), and determine the necessity to incorporate it for staging.

**Methods:**

A total of 809 cases of biopsy‐proven, non‐metastatic CC were identified from an academic cancer center. Recursive partitioning analysis (RPA) method was used to develop the refined staging systems with respect to overall survival (OS). Internal validation was performed by using calibration curve with 1000 bootstrap resampling. Performances of the RPA‐refined stages were compared against the conventional FIGO 2018 and 9th edition TNM‐stage classifications by the receiver operating characteristic curve (ROC) and decision curve analysis (DCA).

**Results:**

We identified that CUI was independently prognostic for death and relapse in our cohort. RPA modeling using a two‐tiered stratification by CUI (positive and negative) and FIGO/T‐categories divided CC into three risk groupings (FIGO I′‐III'/T1′‐3′), with 5‐year OS of 90.8%, 82.1%, and 68.5% for proposed FIGO stage I′–III', respectively (*p* ≤ 0.003 for all pairwise comparisons), and 89.7%, 78.8%, and 68.0% for proposed T1′‐3′, respectively (*p* < 0.001 for all pairwise comparisons). The RPA‐refined staging systems were well validated with RPA‐predicted OS rates showed optimal agreement with actual observed survivals. Additionally, the RPA‐refined stages outperformed the conventional FIGO/TNM‐stage with significantly higher accuracy of survival prediction (AUC: RPA‐FIGO vs. FIGO, 0.663 [95% CI 0.629–0.695] vs. 0.638 [0.604–0.671], *p* = 0.047; RPA‐T vs. T, 0.661 [0.627–0.694] vs. 0.627 [0.592–0.660], *p* = 0.036).

**Conclusion:**

CUI affects the survival outcomes in patients with CC. Disease extended to corpus uterine should be classified as stage III/T3.

## 1 INTRODUCTION

The International Federation of Gynecology and Obstetrics (FIGO) and the American Joint Committee on Cancer (AJCC)/Union for International Cancer Control (UICC) TNM staging system are widely accepted as the lingua franca to depict tumor extent in cervical cancer (CC).^1^, ^2^ These anatomically based FIGO/TNM stage classifications are momentous for treatment decision‐making, risk stratification, response evaluation, prognosis prediction, and facilitating communication among different institutions.^3^, ^4^ Hence, it is critical that the FIGO/TNM classifications are clear and reproducible for tumor evaluation and staging. Nonetheless, the current edition FIGO/TNM staging systems are deemed increasingly inadequate for clinical utility in CC, as they largely determined by clinical evaluation, namely physical examination, which may not accurately reflect the degree of tumor extension.^5^, ^6^ Particularly, both of FIGO and TNM staging systems disregard extension to the uterine corpus, although it had been demonstrated that corpus uteri invasion represents aggressive tumor biological behavior in CC.^7^, ^8^, ^9^ Hence, it still remains controversial whether corpus uteri invasion should be included in the staging system for further prognostication in CC.

Recently, several studies have illustrated that corpus uteri invasion stands for adverse prognostic factor with high risk of distant dissemination and poor survival outcomes in patients with early stage CC.^10^, ^11^, ^12^, ^13^ Additionally, researches have shown that CC had unique pattern of extra‐cervical invasion, namely spreads locally through the embryonic developmental structures; in addition, tumor extend into the uterine corpus is considered to be associated with distinct phenotypic changes of the tumor, which may be related to more aggressive biological behavior of CC.^14^, ^15^, ^16^ These evidence demonstrated that corpus uteri invasion is complementary to conventional FIGO/TNM‐staging for clinical prognostication, and provides additional information that is disregarded by the current classifications. Hence, it is feasible and necessary to include corpus uteri invasion into the staging systems for more precise of tumor evaluation and prognostication. Nonetheless, despite its potential prognostic significance, novel prognostic tools integrating corpus uteri invasion for CC are still not yet developed partly because of the limited sample size of these studies, and lack of appropriate diagnostic and pathological criteria for uterine corpus invasion.^10^, ^11^, ^12^, ^13^ To that end, we hence undergone this study by assembling a well‐characterized dataset of 809 patients who were treated at an academic center, and aiming to construct robust new staging systems by combing corpus uteri invasion with the current FIGO/TNM categories that outperformed the current classifications for prognostication.

## 2 MATERIALS AND METHODS

### 2.1 Patient selection

**FIGURE 1:**
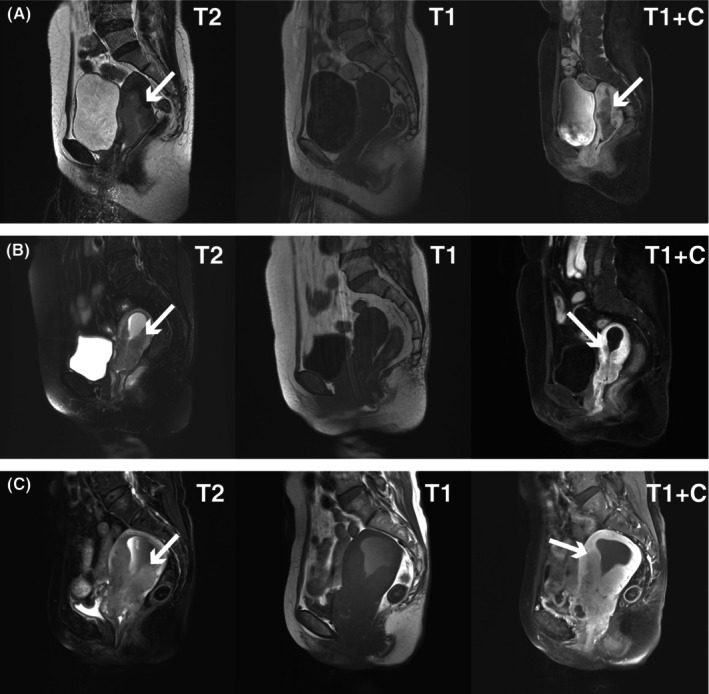
Pelvic MRI images showing uterine body invasion of the cervical cancer patients. (A–C) Uterine body invasion (white sick arrows) shown in the pelvic MRI images of three cervical cancer patients, with an intermediate‐intensity signal on sagittal T2‐weighted images, low‐intensity signal on sagittal T1‐weighted images, and intermediate reinforcing signal on sagittal T1‐enhanced images.

From May 2009 to May 2015, a total of 809 consecutive patients with newly diagnosed, pathology‐proven, FIGO stage I–III CC were screened from a CC‐specific database within the big‐data intelligence platform at the Sun Yat Sen University Cancer Centre (SYSUCC). As we presented previously, the big‐data intelligence platform is a real‐time updated research system that enables real‐time organizing, integrating, and updating of medical records automatically from a number of clinical business systems based on a well‐designed data model and algorithm.^17^ Clinical information including disease description, treatment, and outcomes was obtained from intelligence platform. All patients underwent complete pre‐treatment evaluations including gynecological examination, electrocardiography, pelvic magnetic resonance imaging (MRI), abdominal computed tomography (CT), chest radiography or CT, and whole‐body fluorodeoxyglucose positron emission tomography‐CT (PET/CT), CBC count, biochemical profile, gynecologic tumor markers, and plasma squamous cell carcinoma antigen (SCCA). Uterine body invasion was diagnosed through pelvic MRI, with signs showing a intermediate‐intensity signal on T2‐weighted images, low‐intensity signal on T1‐weighted images, and intermediate reinforcing signal on T1‐enhanced images (Figure 1). All patients were restaged into I/II/III and T1/T2/T3 by two oncologists specializing in gynecological oncology according to the 2018 edition of the FIGO and 9th edition of TNM staging systems, based on the pre‐treatment assessments including clinical, imaging, and pathological findings, with disagreements resolved by consensus. This study was reviewed and approved by the Institutional Review Board at Sun Yat‐sen University Cancer Center (IRB reference No.: 2022‐234‐01).

### 2.2 Treatment and follow‐up

All patients were treated according to the institutional management guidelines for CC. Generally, radical surgery ± adjuvant radiotherapy with or without chemotherapy was proposed for the patients with FIGO stage I‐IIA, and EBRT + concurrent platinum‐containing chemotherapy + brachytherapy was recommended for those with FIGO stage IIB‐III. Reasons for deviation from the guidelines included recruitment in clinical trials, individual patient's refusal, age, or organ dysfunction suggestive of intolerance to treatment.

After treatment, patients were required to follow‐up at 3‐month intervals for the first 2 years, every 6 months for 3–5 years, and then annually thereafter or until death. At each follow‐up visit, interval H&P, plasma SCCA, and patient education regarding symptoms of potential recurrence, periodic self‐examinations, lifestyle, obesity, exercise, sexual health, and potential long‐term and late effects of treatment were routinely performed. Imaging examinations including pelvic MRI, abdominal CT, or MRI, chest radiography, or CT were performed every 6 months for the first 2 years, and then annually thereafter, or when clinically implying of tumor relapse. The primary endpoint was overall survival (OS), which was calculated as the duration from the date of diagnosis to death from any cause, or to date of last follow‐up visit. The secondary endpoints were progression‐free survival (PFS), calculated as the duration from the date of diagnosis to the first evidence of tumor progress regardless of site or death from any cause or date of last follow‐up visit; distant metastasis‐free survival (DMFS), from the date of diagnosis to the first evidence of distant metastasis and/or death.

### 2.3 Statistical analysis

Categorical variables were compared using the chi‐squared or Fisher's exact test. Considering that multiple treatment modalities were used in this cohort, we classified treatment as the following groups: chemo‐radiotherapy (CRT), radiotherapy (RT), surgery (S), and S+adjuvant therapy (S+A). The Kaplan–Meier method was performed for the estimation of cumulative survival rates, and survival curves were compared using the log‐rank test. Cox proportional hazards regression was used for calculation of hazard ratio (HR) with 95% confidence interval (CI) in univariate and multivariable analyses.

To evaluate the role of corpus uteri invasion for prognostication and construct new staging systems that incorporating 2018 FIGO/9th edition T‐category and corpus uteri status, we conducted the recursive partitioning analysis (RPA) with respect to OS to drive new RPA stage groupings in this cohort. The RPA algorithm is based on the optimized binary partition of FIGO categories and corpus uteri status, and results in subgroups with relatively homogeneous survival performance. Validation of the proposed RPA staging system was performed by calibration curves comparing RPA‐predicted versus observed Kaplan–Meier estimates of survival probability, and a bootstraps with 1000 resamples was applied in this process. Additionally, we performed the receiver operating characteristic (ROC) curve and decision curve analysis (DCA) to evaluate the accuracy and efficacy of RPA staging system for survival prediction against 2018 FIGO classification. All statistical tests were two‐sided, and a *p*‐value of <0.05 was considered significant. Statistical analyses were performed in R version 3.4.4 (http://www.r‐project.org/), and SPSS 23.0 software (SPSS Inc, IL).

## 3 RESULTS

### 3.1 Patient demographics and survival outcomes

Clinical characteristics of the patients are listed in Table 1; corpus uteri invasion was identified in 18.7% (151 of 809) of patients. With median follow‐up duration of 71.6 ([IQR] 45.8–86.2) months, we observed 102 cases of distant metastasis and 68 cases of loco‐regional relapse, respectively; estimated 5‐year OS, DMFS, and PFS rates were 80.9% (95% confidence interval [CI] 79.5%–82.3%), 79.4% (95% CI 77.9%–80.9%), and 74.8% (95% CI 73.2%–76.4%), respectively.

**TABLE 1:** General characteristics.

Characteristic	All (*n* = 809)	Corpus uteri negative (*n* = 658)	Corpus uteri positive (*n* = 151)	*p*‐value
Age (years)				
Median	50	49	52	0.002
IQR	42–57	41–56	46–58	
Smoking, *n* (%)				
None	801 (99.0)	652 (99.1)	149 (98.7)	0.647
Yes	8 (1.0)	6 (0.9)	2 (1.3)	
Drinking, *n* (%)				
None	800 (98.9)	651 (98.9)	149 (98.7)	0.678
Yes	9 (1.1)	7 (1.1)	2 (1.3)	
WHO histologic type, *n* (%)				
SCC	674 (83.3)	548 (83.3)	126 (83.4)	0.384
AC	93 (11.5)	79 (12.0)	14 (9.3)	
ASC	19 (2.3)	13 (2.0)	6 (4.0)	
Other	23 (2.8)	18 (2.7)	5 (3.3)	
T category, *n* (%)				
T1	380 (47)	345 (52.4)	35 (23.2)	<0.001
T2	358 (44.2)	274 (41.7)	84 (55.6)	
T3	71 (8.8)	39 (5.9)	32 (21.2)	
FIGO 2018, *n* (%)				
I	335 (41.4)	310 (47.1)	25 (16.6)	<0.001
II	263 (32.5)	219 (33.3)	44 (29.1)	
III	211 (26.1)	129 (19.6)	82 (54.3)	
Treatment, *n* (%)				
CRT	115 (14.2)	75 (11.4)	40 (26.5)	<0.001
RT	57 (7.0)	34 (5.2)	23 (15.2)	
S	166 (20.5)	159 (24.2)	7 (4.6)	
S+A	471 (58.2)	390 (59.3)	81 (53.6)	

Abbreviations: A, adjuvant therapy; AC, Adenocarcinoma; ASC, Adenosquamous Carcinoma; CRT, chemo‐radiotherapy; IQR, interquartile range; NA, not available; RT, radiotherapy; S, surgery; SCC, Squamous Cell Carcinoma.

### 3.2 Effect of corpus uteri invasion on prognostication

In the whole cohort, we observed that patients with corpus uteri invasion exerted significantly more advanced stage (corpus uteri positive vs. negative: FIGO stage III, 54.3% vs. 19.6%, *p* < 0.001; T3, 21.2% vs. 5.9%, *p* < 0.001; Table 1). Additionally, corpus uteri invasion was demonstrated to be an adverse prognostic factor for death and/or disease relapse (*p* < 0.001 for all comparisons, Figure S1); patients with corpus uteri invasion had significantly inferior OS (5 year rate: corpus uteri positive vs. negative, 66.7% [95% CI 62.7%–70.7%] vs. 84.2% [82.7%–85.7%], *p* < 0.001; Table S1), PFS (61.1% [57.0%–65.2%] vs. 79.2% [77.6%–80.8%], *p* < 0.001; Table S1), and DMFS (61.3% [57.2%–65.4%] vs. 83.6% [82.1%–85.1%], *p* < 0.001; Table S1) than those without corpus uteri invasion. Furthermore, multivariable analyses also identified that corpus uteri invasion was independently adverse significant for OS, PFS, and DMFS (*p* < 0.001 for all, Table S2).

**FIGURE 2:**
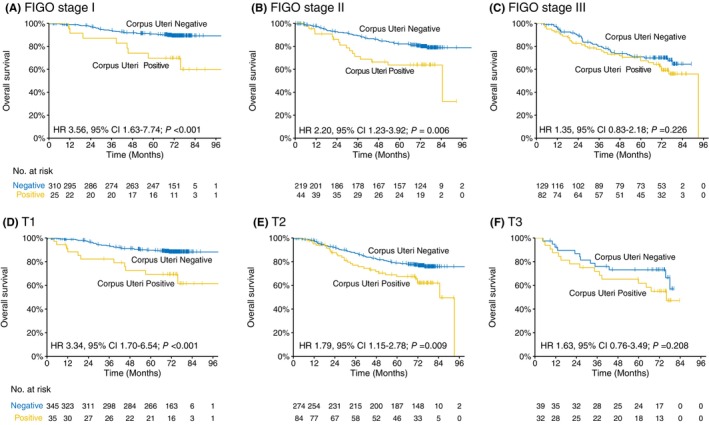
Kaplan–Meier curves of overall survival for the positive and negative corpus uteri invasion within each FIGO stage and T category. Corpus uteri invasion was adverse for OS in FIGO stage I‐II (A, B) and T1‐2 (D, E), but not in stage III (C) and T3 (F).

Next, to investigate the potential interaction effect between corpus uteri invasion and FIGO 2018 stage/9th edition T‐category on prognostication, we then performed subgroup analysis by exploring the prognostic effect of corpus uteri invasion within each FIGO stage and T category. As shown in Figure 2, corpus uteri invasion was adverse for OS in FIGO stage I‐II (HR 3.56 [95% CI 1.63–7.74], *p* < 0.001 in stage I; and 2.20 [1.23–3.92], *p* = 0.006 in stage II; Figure 2A,B) and T1‐2 (HR 3.34 [95% CI 1.70–6.54], *p* < 0.001 in T1; and 1.79 [1.15–2.78], *p* = 0.009 in T2; Figure 2D,E), but not in stage III (HR 1.35 [95% CI 0.83–2.81], *p* = 0.226; Figure 2C) and T3 (HR 1.63 [95% CI 0.76–3.49], *p* = 0.208; Figure 2F). We therefore conclude that corpus uteri invasion is a critical risk factor for prognostication in early stage CC (FIGO stage I‐II and T1‐2), but did not have impact in advanced stage disease (FIGO stage III and T3).

### 3.3 Development of refined staging systems using corpus uteri status and FIGO 2018 classification and 9th edition TNM schema

To investigate the role of corpus uteri invasion for staging in CC and finally develop refined staging systems that incorporate corpus uteri status and the current staging classifications (FIGO 2018 and 9th edition TNM schema), we first tested the prognostic value of corpus uteri invasion for death and/or disease relapse (Figure S2). Interestingly, we observed that the prognosis of patients with corpus uteri invasion within FIGO stage I‐II CC was close to FIGO stage III in terms of OS, PFS, and DMFS, which was significantly inferior than those without corpus uteri invasion (Figure S2A). This was also identified between corpus uteri invasion and T‐category (Figure S2C).

**FIGURE 3:**
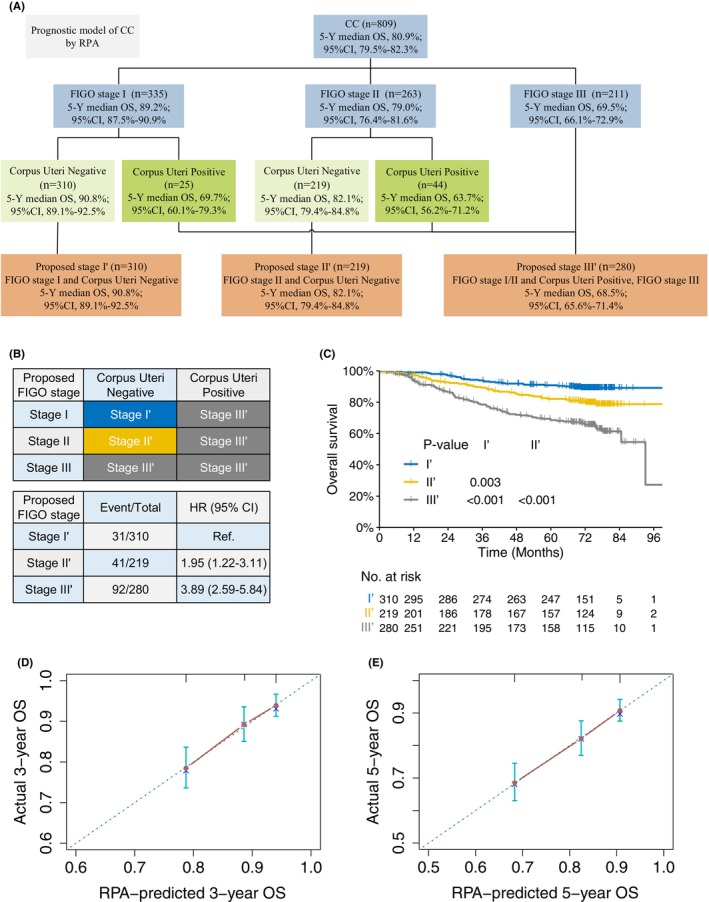
Proposed RPA‐FIGO staging system incorporating the corpus uteri status for M0 cervical cancer. Prognostic model for M0 cervical cancer by RPA method combing corpus uteri status and the 2018 FIGO categories. (B) Risk groups derived by the proposed RPA‐FIGO classification system and the corresponding distribution of patients, compared against the 2018 FIGO staging system. (C) Kaplan–Meier curves for OS stratified by the RPA‐FIGO risk classifications. (D) Calibration plots illustrated that the RPA‐predicted 3‐ and 5‐year OS rates had optimal agreement with actual observed survivals.

**FIGURE 4:**
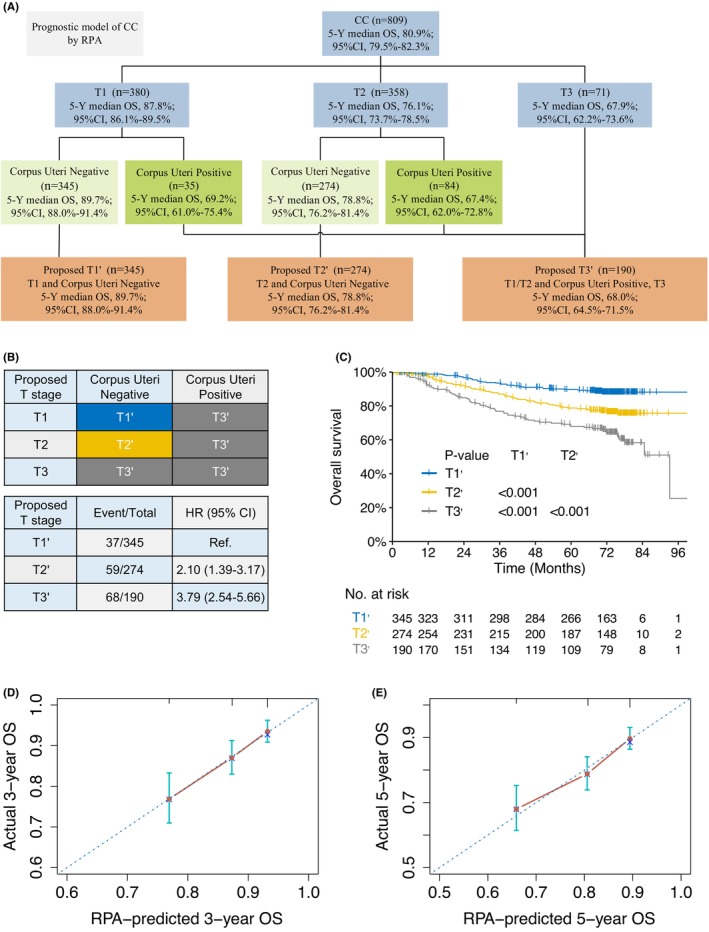
Proposed RPA‐T staging system incorporating the corpus uteri status for M0 cervical cancer. Prognostic model for M0 cervical cancer by RPA method combing corpus uteri status and the 9th TNM stage schema. (B) Risk groups derived by the proposed RPA‐T classification system and the corresponding distribution of patients, compared against the 9th TNM staging system. (C) Kaplan–Meier curves for OS stratified by the RPA‐T risk classifications. (D) Calibration plots illustrated that the RPA‐predicted 3‐ and 5‐year OS rates had optimal agreement with actual observed survivals.

To this end, we constructed two new staging systems combining corpus uteri status and the current stage systems (2018 FIGO categories and 9th TNM stage schema, respectively), using the RPA modeling method, which resulted in two new risk classification systems that both contain three stage groupings with FIGO stage I‐II and T1‐2 being upgraded to a higher RPA risk based on corpus uteri invasion: proposed stage I′ (T1′), FIGO stage I (or T1) and corpus uteri negative; proposed stage II' (T2′), FIGO stage II (or T2) and corpus uteri negative; and proposed stage III' (T3′), FIGO stage III (or T3) or corpus uteri positive (Figure 3A and 4A). General clinical characteristics of the patients within each proposed RPA stage groups are illustrated in Table S3. Our RPA‐based stage groupings demonstrated good performance for hazard discrimination with significant separated risks for death and/or disease relapse (Figures 3B and 4B; Figure S2) and disparate survival outcomes (Figures 3C and 4C and Figure S3); with 5‐year OS of 90.8%, 82.1%, and 68.5% for proposed FIGO stage I′–III' respectively (*p* ≤ 0.003 for all pairwise comparisons, Figure 3C), and 89.7%, 78.8%, and 68.0% for proposed T1′‐3′ respectively (*p* < 0.001 for all pairwise comparisons, Figure 4C). Additionally, our proposed RPA staging systems had good performance for survival prediction; the calibration plots illustrated that the RPA‐predicted 3‐ and 5‐year OS rates had optimal agreement with actual observed survivals (Figures 3D,E and 4D,E).

### 3.4 Performances of the proposed RPA‐based staging systems against conventional FIGO and TNM classifications

Next, we compared the proposed RPA staging systems against the 2018 FIGO classification and 9th edition TNM schema. Table 2 summarizes the area under ROC curves (AUCs) of the refined RPA staging systems and conventional stage schemas. The RPA‐refined staging system outperformed the conventional stage schemas with significantly higher accuracy for survival prediction in terms of OS (AUC: RPA‐FIGO vs. FIGO, 0.663 [95% CI 0.629–0.695] vs. 0.638 [0.604–0.671], *p* = 0.047; RPA‐T vs. T, 0.661 [0.627–0.694] vs. 0.627 [0.592–0.660], *p* = 0.036), PFS (RPA‐FIGO vs. FIGO, 0.652 [0.618–0.685] vs. 0.622 [0.587–0.655], *p* = 0.015; RPA‐T vs. T, 0.643 [0.608–0.676] vs. 0.609 [0.574–0.643], *p* = 0.024) and DMFS (RPA‐FIGO vs. FIGO, 0.662 [0.628–0.694] vs. 0.620 [0.585–0.653], *p* = 0.004; RPA‐T vs. T, 0.652 [0.618–0.684] vs. 0.601 [0.567–0.635], *p* = 0.004) (Table 2 and Figure S4 and S5). Additionally, DCA also confirmed that RPA‐refined staging systems were superior to conventional FIGO and T classifications with higher efficacy for prognostication (Figures S4 and S5). In multivariable analyses, RPA‐refined stages were both independent significant predictors for OS (*p* ≤ 0.003 for all), PFS (*p* ≤ 0.003 for all), and DMFS (*p* ≤ 0.014 for all) (Table S4). We therefore conclude that our RPA staging system was the optimal risk classification system for CC.

**TABLE 2:** Comparing of AUC of RPA staging systems with the 2018 FIGO/9th edition T classifications.

	Overall survival			Progression‐free survival			Distant metastasis‐free survival		
	AUC	95% CI	*p*‐value	AUC	95% CI	*p*‐value	AUC	95% CI	*p*‐value
RPA_T	0.661	0.627–0.694	Ref.	0.643	0.608–0.676	Ref.	0.652	0.618–0.684	Ref.
T category	0.627	0.592–0.660	**0.036**	0.609	0.574–0.643	**0.024**	0.601	0.567–0.635	**0.004**
Corpus Uteri	0.597	0.562–0.631	**<0.001**	0.584	0.550–0.619	**0.002**	0.604	0.569–0.637	**0.004**
RPA_FIGO	0.663	0.629–0.695	Ref.	0.652	0.618–0.685	Ref.	0.662	0.628–0.694	Ref.
FIGO 2018	0.638	0.604–0.671	**0.047**	0.622	0.587–0.655	**0.015**	0.620	0.585–0.653	**0.004**
Corpus Uteri	0.597	0.562–0.631	**<0.001**	0.584	0.550–0.619	**<0.001**	0.604	0.569–0.637	**0.003**

Bold values were statistically significant (< 0.05).

Abbreviations: AUC, the area under the receiver operating characteristic curve; CI, confidence interval.

Finally, we presented the clinical impact of our RPA‐refined staging systems against the existing 2018 FIGO and 9th edition T classifications (Table S5). Our RPA staging schemas were able to re‐classify patients from early FIGO‐stage/T‐category group in CC, thereby highlighting the great impact of corpus uteri invasion on prognostication and intra‐group heterogeneity by the current stage classification systems.

## 4 DISCUSSION

Conventional FIGO and TNM‐stage classification represent sound systems for the clinical risk stratification of patients to inform on prognosis and decision‐making for CC. Nonetheless, it is confined by the simplistic consideration of primary and regional tumor burden based on physical examination and limited imaging modalities, which may not exactly reflect the degree of tumor extent and are not complete applicable to clinical practice. Consequently, several important prognostic indicators such as corpus uteri invasion are still not included in the current FIGO and TNM staging classifications. To address this unmet need, we adopted a big‐data approach by assembling a large‐scale, well‐characterized dataset of 809 cases of CC, all of whom had pre‐treatment image evaluation and diagnostic staging that were centrally performed. We identified that corpus uteri invasion was independently adverse prognostic for death and disease relapse in CC and had obvious interaction with FIGO stage and T classification. We then applied an intuitive two‐tiered classification schema to integrate corpus uteri status with conventional FIGO stage and T‐category using the RPA modeling method, and divided patients into three risk groupings that are more homogeneous in terms of risk of death within each subgroup. We also identified that our proposed RPA staging systems outperformed the FIGO‐stage and T‐category for prognostication with most superior accuracy for prediction of death and disease relapse. Additionally, our RPA models were well validated in calibration plots illustrating optimal agreement of the RPA‐predicted OS rates with actual observed survivals. Based on these findings, we have confirmed the role of corpus uteri invasion for staging in CC, and presented two new staging systems incorporating conventional FIGO‐stage and T‐category with baseline corpus uteri status for CC that outperform the existing stage classification systems using the large‐scale well‐characterized dataset.

Corpus uteri invasion in CC is well recognized as an important indicator of tumor aggressiveness and a predictor of poor survival outcome. The current study illustrated that the poor prognosis of corpus uteri invasion in CC was due to the significant increased risk of distant metastasis, rather than loco‐regional relapse (Appendix Table S6 and Figure S6); this was keep in accordance with the results of previous reports.^18^, ^19^ A reasonable explanation for the tendency of distant dissemination of corpus uteri invasion is that the myometrium has a wide distribution of blood vessels and lymphatic network, which facilitate tumor cell lymphatic and hematogenous dissemination from cervix to pelvic and/or para‐aortic lymph nodes and distant organs.^20^ Additionally, recent studies have shown that CC spreads locally through the embryonic developmental structures within the utero‐vaginal compartment derived from the Müllerian anlage, and tumor spread beyond this anatomical barrier such as uterine corpus invasion is considered a late event which associated with distinct phenotypic changes of the tumor, as the compartment borders acts as a tumor suppressor, functioning to contain tumor and preventing extension beyond this barrier.^15^, ^16^ Hence, it is rational to speculate that the anatomic characteristics of corpus uteri and molecular alterations of tumor cells contributed to the differing tumor spread patterns of CC.

Additionally, considering the perennial issues whether corpus uteri invasion should be included for staging of CC, the proposed RPA‐derived staging systems afford intuitive approaches for incorporation of corpus uteri invasion within the FIGO/T stages. In the current study, we identified that uterine corpus invasion was robust for risk discretization for early stage (FIGO I‐II and/or T1‐2) CC. The prognosis of patients with corpus uteri invasion within early stage (FIGO I‐II/T1‐2) was significantly inferior than those without corpus uteri invasion, whereas was close to the locally advanced diseases (FIGO III/T3). Hence, it is convictive that early stage disease with corpus uteri invasion should be upgraded to a higher staging group, and our proposed RPA‐derived models provide compelling solutions for better prognostication in CC. More importantly, the RPA‐derived staging systems were superior over the conventional FIGO/T stage classifications with better performances in terms of survival prediction. Contrary to extensive use of parametrial and vaginal involvement in the existing staging systems,^1^, ^2^ the proposition to incorporate corpus uteri invasion for prognostication in CC is contentious for several reasons. Nonetheless, some gynecologic oncologists still argue that, patients with invasion of the corpus uterine (assuming all remaining findings in the pelvis negative for disease) should be allocated as locally advanced stage, given that the corpus uterine is part of an adjacent pelvic organ and uterine corpus invasion had been shown to be associated with poor prognosis and increased risk of lymph node metastasis.^8^ The current study also indicate that corpus uteri invasion combined with FIGO/T stages conduce to improvement of the accuracy of prognosis prediction in CC.

Our results support the use of MRI for the detection of corpus uteri invasion in CC. The uterine corpus is a cavity smooth muscular organ, which consists of serous membrane, muscularis, and mucous membrane (intima). MRI is an ideal technique in the assessment of normal anatomic tissue characterization and abnormalities of uterine corpus with excellent soft‐tissue contrast, which facilitates to clearly depict the differential zonal anatomy of the corpus uteri and the cyclical endometrial changes during the menstrual cycle as well as malignancies within the uterus.^21^ Compared to other imaging modalities and pathological diagnosis, MRI examination is non‐radioactive, non‐invasive and has advantages to demonstrate blood vessels without the use of intravenous contrast, as well as obtaining information on uterine corpus invasion prior to treatment. Previous researches have illustrated that pre‐treatment MRI is sensitive to detect corpus uteri invasion in CC,^22^ and image‐detected uterine corpus tumor invasion is indeed correlated to outcome in women with advanced CC. Hence, considering the superiority of MRI in assessment of local disease extent, the National Comprehensive Cancer Network (NCCN) guidelines has recommended pelvic MRI with contrast for initial workup of CC. Similarly, MRI has been proposed as the gold standard for tumor staging in the head and neck cancers, for example, nasopharyngeal carcinoma.

There were several caveats of our research ought to be highlighted. Foremost, we did not investigate the association of our proposed RPA staging groups with efficacy of different treatment modalities, as interventions are not baseline attributes, and this needs to be verified by prospective clinical trials, where control (e.g., randomization) over the assignment of treatments were performed to investigate the appropriate treatment strategy for each RPA risk group. Additionally, this analysis is beyond the scope of this study, especially given the potential physician bias in treatment recommendation for the patients. For all this, corpus uteri invasion was associated with significant higher risk of distant metastasis, thus systemic intensification should be proposed for these patients. Next, this is limited by its retrospective study nature, and patients recruited from a single institution; hence, the results of our study need to be validated in prospective dataset from other centers.

In conclusion, we identified that corpus uteri invasion was an effective indicator for risk stratification and prognostication in CC. Additionally, we successfully constructed two‐tiered RPA‐based staging systems by combining corpus uteri status with the conventional FIGO/TNM classifications for CC. The new staging schema stratify patients into three stage groupings with disparate survival outcomes, and both outperformed the existing FIGO/TNM staging systems for survival prognostication. Our results indicate that treatment modification for CC was essential, and the proposed RPA‐staging systems could be the basis for future strategies of streamlining treatments in CC patients.

## AUTHOR CONTRIBUTIONS

**Xiaodan Huang:** Conceptualization (equal); data curation (equal); formal analysis (equal); funding acquisition (equal); investigation (equal); methodology (equal); project administration (equal); software (equal); validation (equal); visualization (equal); writing – original draft (lead); writing – review and editing (equal). **Kai Chen:** Conceptualization (equal); data curation (equal); formal analysis (equal); investigation (equal); methodology (equal); project administration (equal); software (equal); validation (equal); visualization (equal); writing – original draft (lead); writing – review and editing (equal). **Liu Shi:** Conceptualization (equal); data curation (equal); formal analysis (equal); investigation (equal); methodology (equal); project administration (equal); software (equal); visualization (equal); writing – original draft (equal); writing – review and editing (equal). **Ying‐Shan Luo:** Conceptualization (equal); data curation (equal); formal analysis (equal); investigation (equal); methodology (equal); project administration (equal); visualization (equal); writing – original draft (equal); writing – review and editing (equal). **Yi OuYang:** Conceptualization (supporting); data curation (supporting); investigation (supporting); resources (equal); writing – original draft (supporting); writing – review and editing (supporting). **JunYun Li:** Data curation (supporting); formal analysis (supporting); investigation (supporting); visualization (supporting); writing – original draft (supporting); writing – review and editing (supporting). **Lan‐Qing Huo:** Data curation (equal); methodology (supporting); software (equal); writing – review and editing (supporting). **Lin Huang:** Data curation (equal); formal analysis (equal); writing – review and editing (equal). **Foping Chen:** Conceptualization (lead); data curation (lead); formal analysis (equal); funding acquisition (equal); investigation (equal); resources (lead); software (equal); supervision (lead); writing – original draft (equal); writing – review and editing (equal). **XinPing Cao:** Conceptualization (lead); data curation (lead); funding acquisition (lead); investigation (equal); resources (lead); software (equal); supervision (lead); writing – original draft (equal); writing – review and editing (equal).

## FUNDING INFORMATION

This study was supported by grants from the National Natural Science Foundation of China (81972430, 82002428 and 82103155) and the China Postdoctoral Science Foundation (2021 M693640). The sponsors had no role in shaping study design, data collection, data analysis, data interpretation, or writing of the report. The corresponding authors had full access to the data and had final responsibility for the decision to submit the manuscript for publication.

## CONFLICT OF INTEREST STATEMENT

The authors declare no conflict of interest. The authenticity of this article has been validated by uploading the key raw data onto the Research Data Deposit public platform (www.researchdata.org.cn), with the approval number as RDDA2022692034.

## Supporting information


Figure S1.

Figure S2.

Figure S3.

Figure S4.

Figure S5.

Figure S6.

Table S1.

## Data Availability

The authenticity of this article has been validated by uploading the key raw data onto the Research Data Deposit public platform (www.researchdata.org.cn).
