# Supplementary figures and images for: Construction of refined staging classification systems integrating FIGO/T‐categories and corpus uterine invasion for non‐metastatic cervical cancer
Source: Cancer Med. 2023 Jun 16;12(14):15079–89. doi: 10.1002/cam4.6179 (PMC10417195; doi:10.1002/cam4.6179)

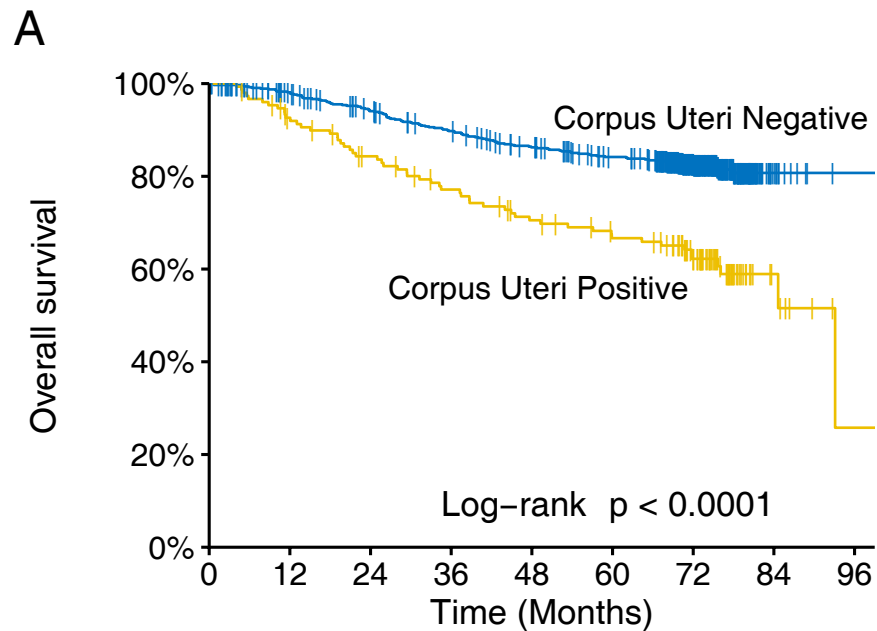

No. at risk

Negative 658 612 574 541 509 477 328 16 3

Positive 151 135 119 106 94 85 62 8 1

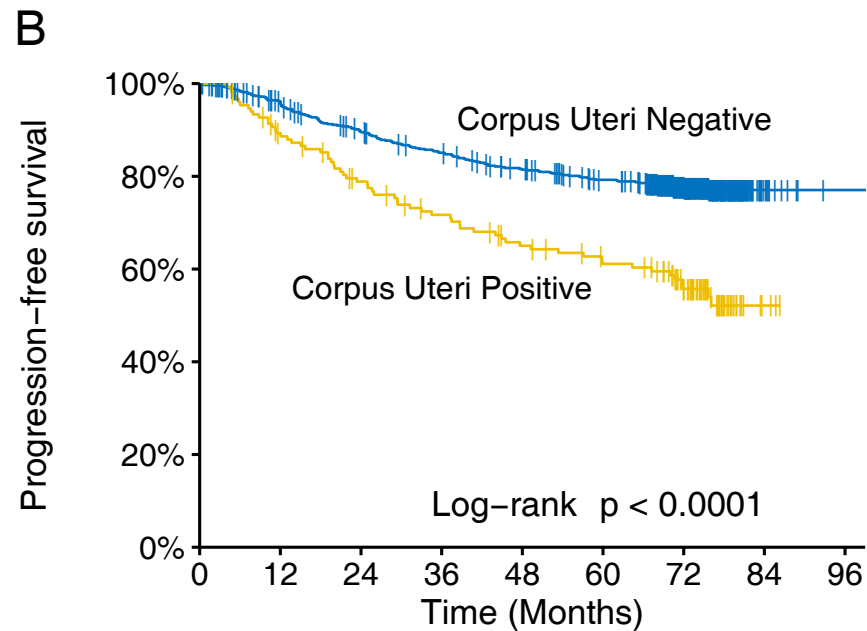

658 601 553 520 489 458 313 16 3

151 130 111 98 86 77 54 3 0

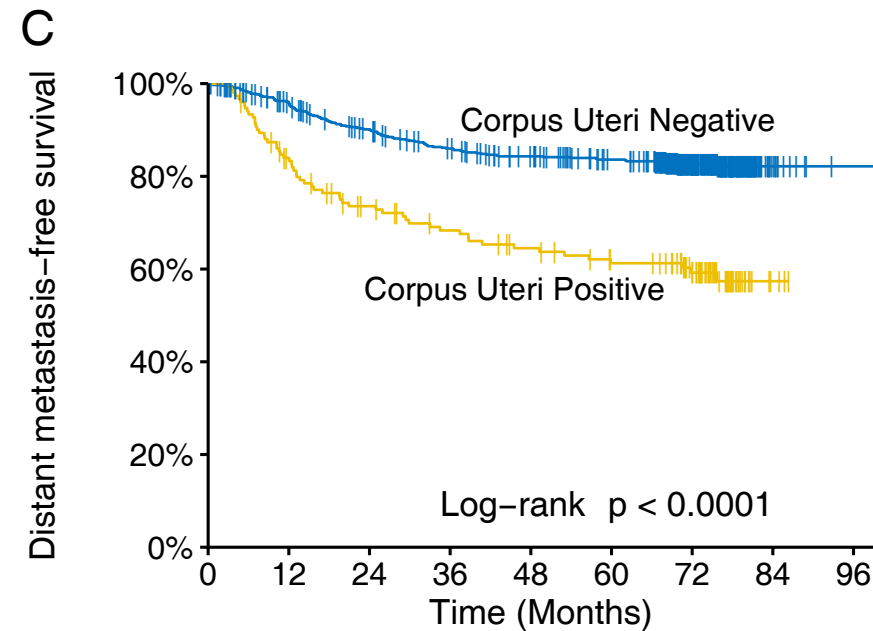

658 600 552 516 491 464 317 16 3

151 122 101 90 82 73 54 3 0

Supplement: Supplementary file 1 — Figure S1. [file CAM4-12-15079-s003.pdf]

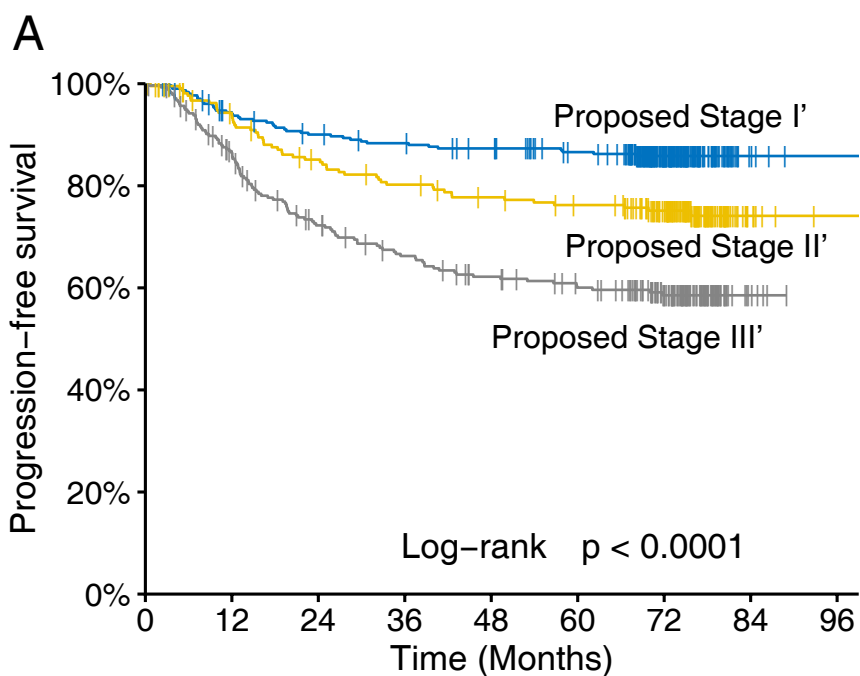

No. at risk

|            |     |     |     |     |     |     |     |   |   |
|------------|-----|-----|-----|-----|-----|-----|-----|---|---|
| Stage I'   | 310 | 281 | 267 | 260 | 253 | 239 | 146 | 5 | 1 |
| Stage II'  | 219 | 195 | 174 | 163 | 155 | 148 | 118 | 9 | 2 |
| Stage III' | 280 | 229 | 184 | 164 | 149 | 138 | 100 | 5 | 0 |

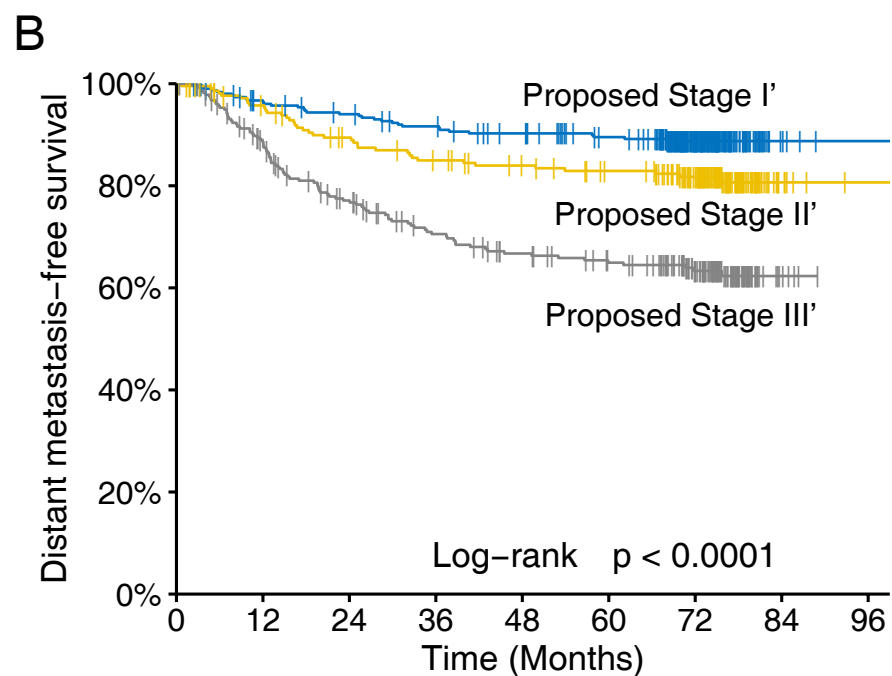

|  |     |     |     |     |     |     |     |   |   |
|--|-----|-----|-----|-----|-----|-----|-----|---|---|
|  | 310 | 288 | 278 | 268 | 258 | 243 | 148 | 5 | 1 |
|  | 219 | 199 | 181 | 170 | 162 | 153 | 121 | 9 | 2 |
|  | 280 | 235 | 195 | 168 | 153 | 141 | 102 | 5 | 0 |

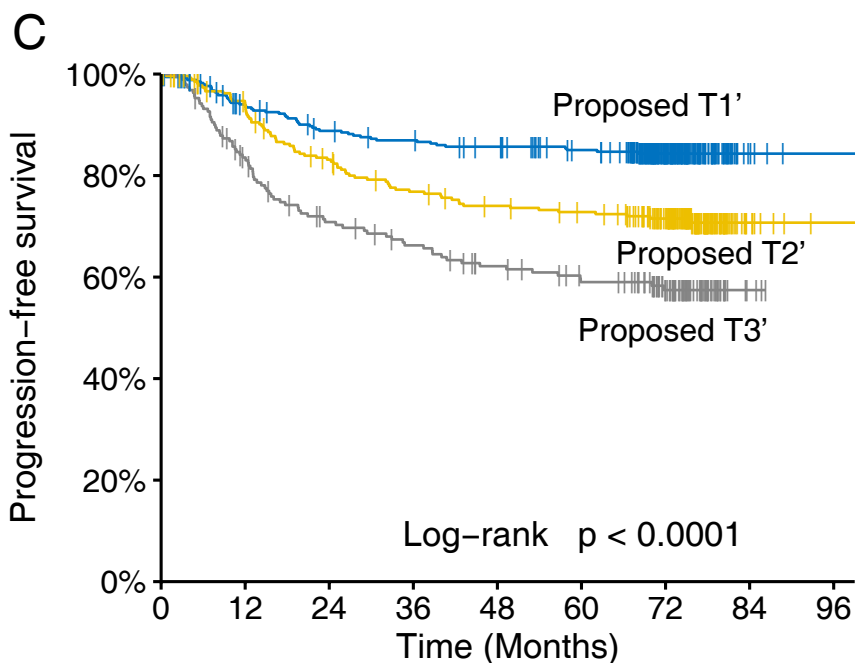

No. at risk

|     |     |     |     |     |     |     |     |    |   |
|-----|-----|-----|-----|-----|-----|-----|-----|----|---|
| T1' | 345 | 307 | 287 | 279 | 271 | 256 | 156 | 6  | 1 |
| T2' | 274 | 245 | 213 | 194 | 184 | 177 | 142 | 10 | 2 |
| T3' | 190 | 153 | 125 | 114 | 102 | 92  | 66  | 3  | 0 |

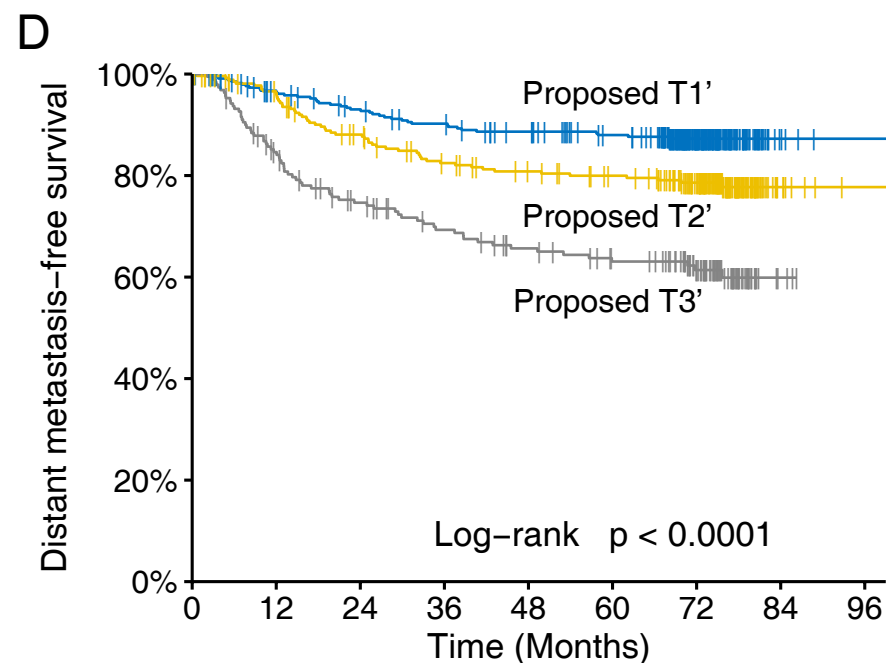

|  |     |     |     |     |     |     |     |    |   |
|--|-----|-----|-----|-----|-----|-----|-----|----|---|
|  | 345 | 316 | 300 | 288 | 276 | 260 | 158 | 6  | 1 |
|  | 274 | 251 | 223 | 203 | 193 | 183 | 145 | 10 | 2 |
|  | 190 | 155 | 130 | 115 | 104 | 94  | 68  | 3  | 0 |

Supplement: Supplementary file 3 — Figure S3. [file CAM4-12-15079-s007.pdf]

A: ROC-OS

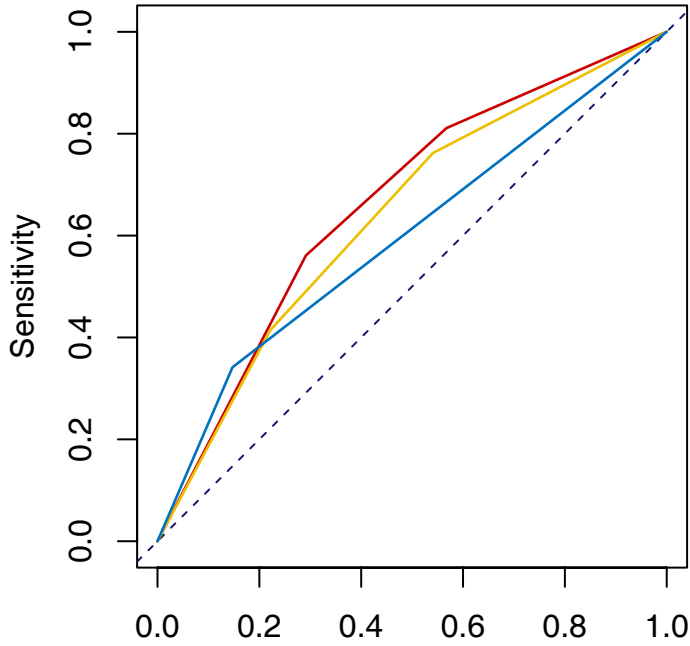

B: ROC-PFS

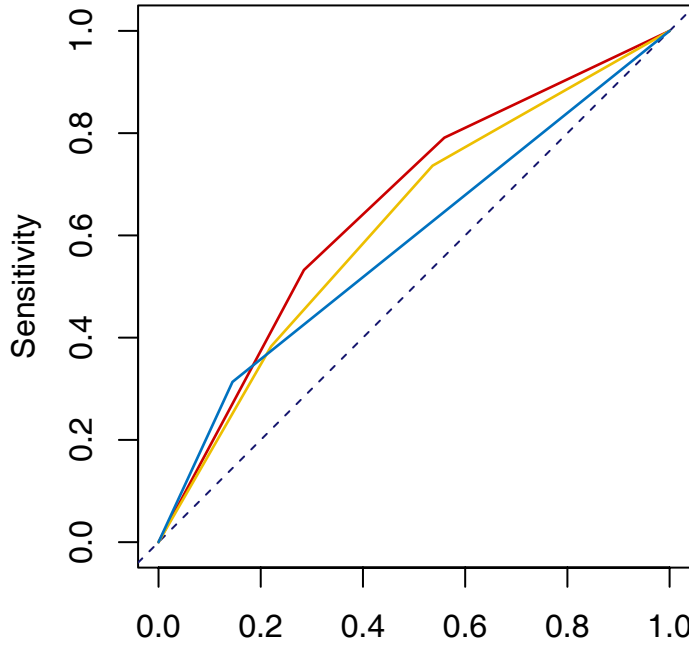

C: ROC-DMFS

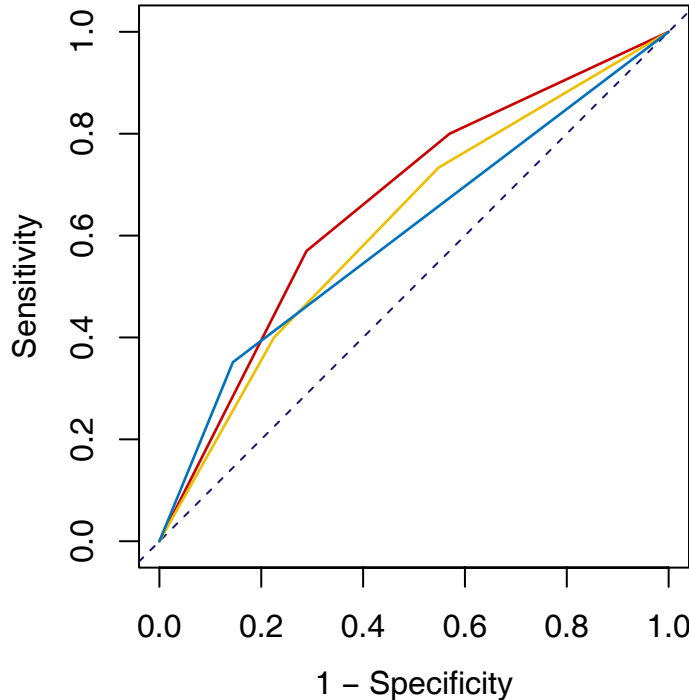

- RPA-FIGO
- FIGO
- Corpus Uteri
- All
- None

D: DCA-OS

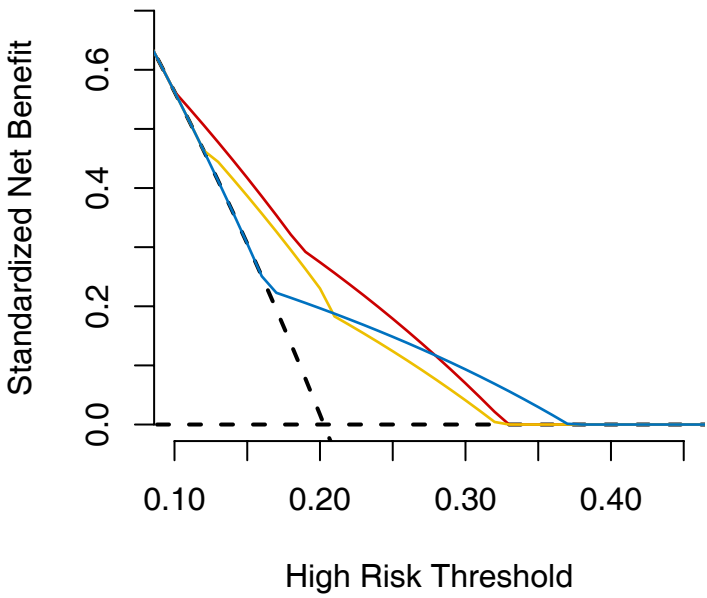

E: DCA-PFS

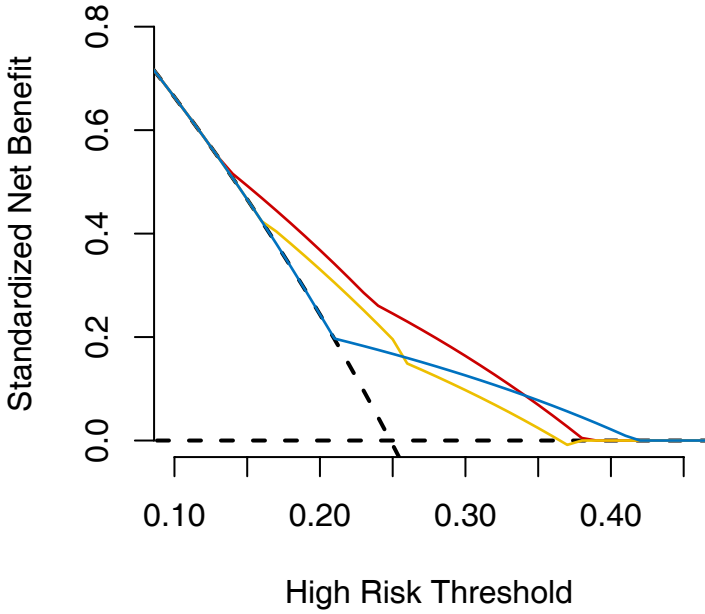

F: DCA-DMFS

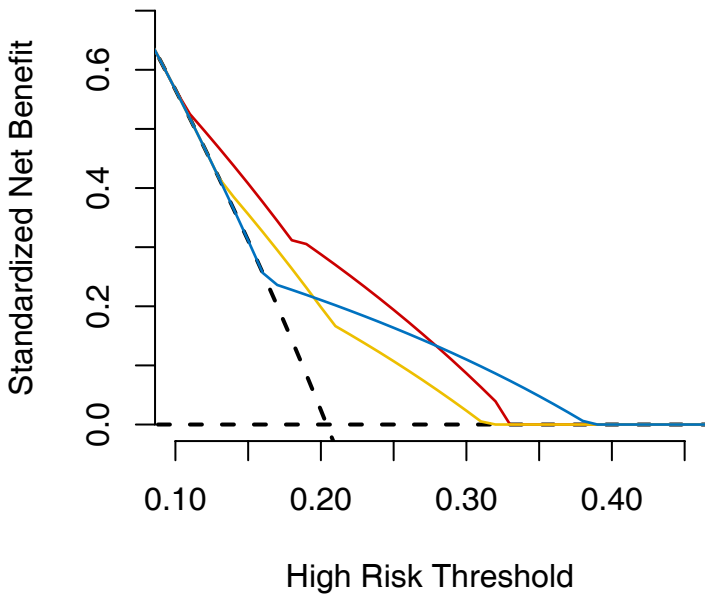

Supplement: Supplementary file 4 — Figure S4. [file CAM4-12-15079-s001.pdf]

A: ROC-OS

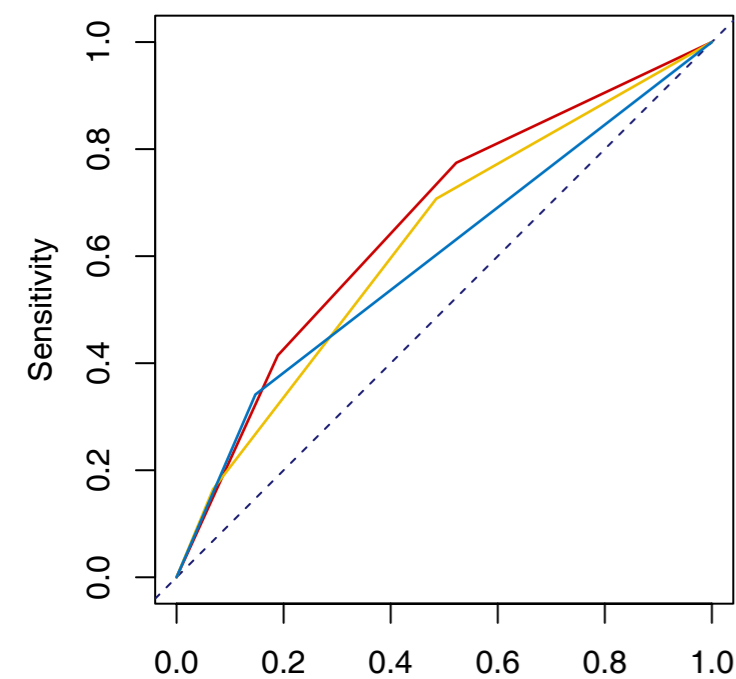

B: ROC-PFS

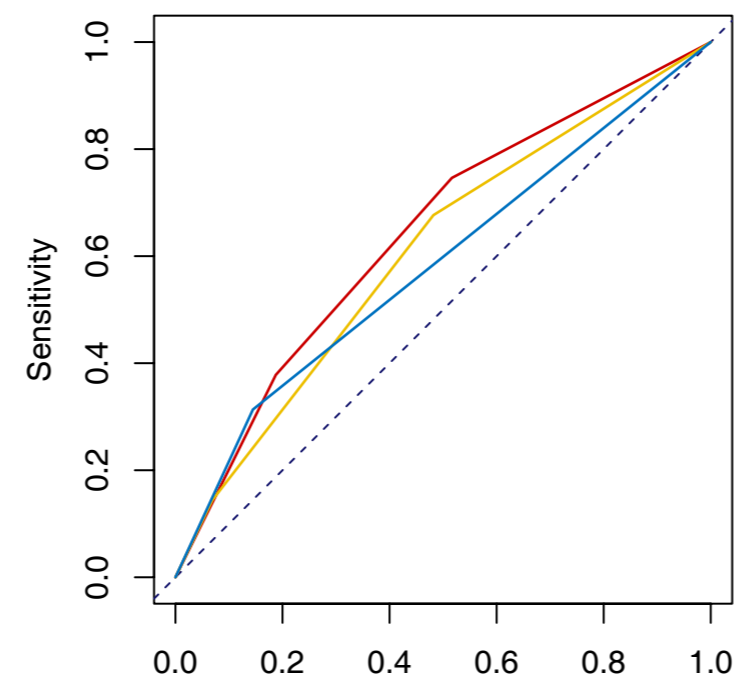

C: ROC-DMFS

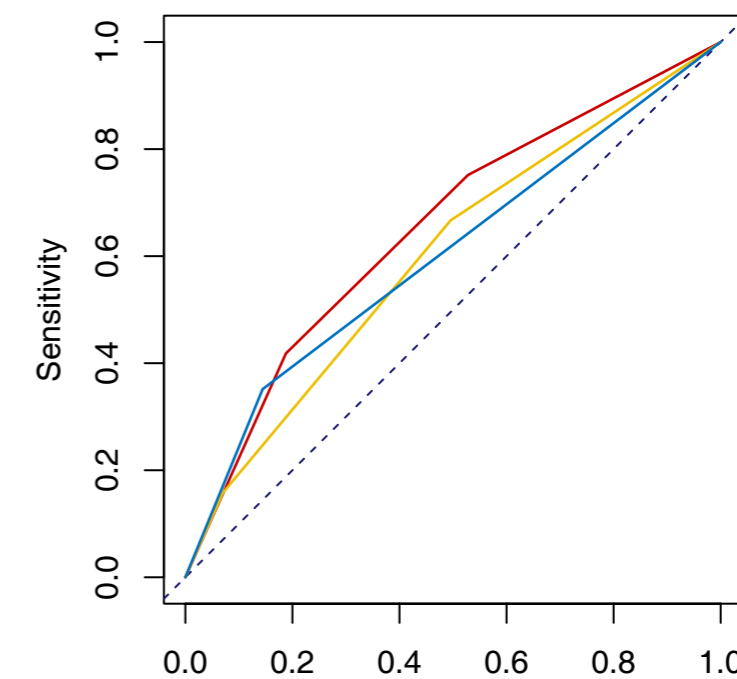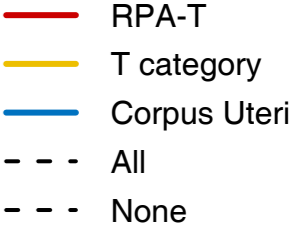

D: DCA-OS

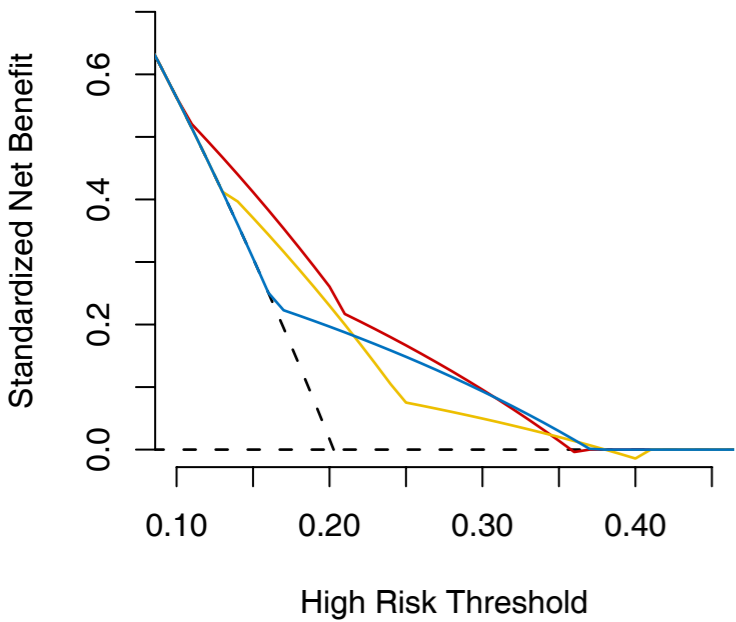

E: DCA-PFS

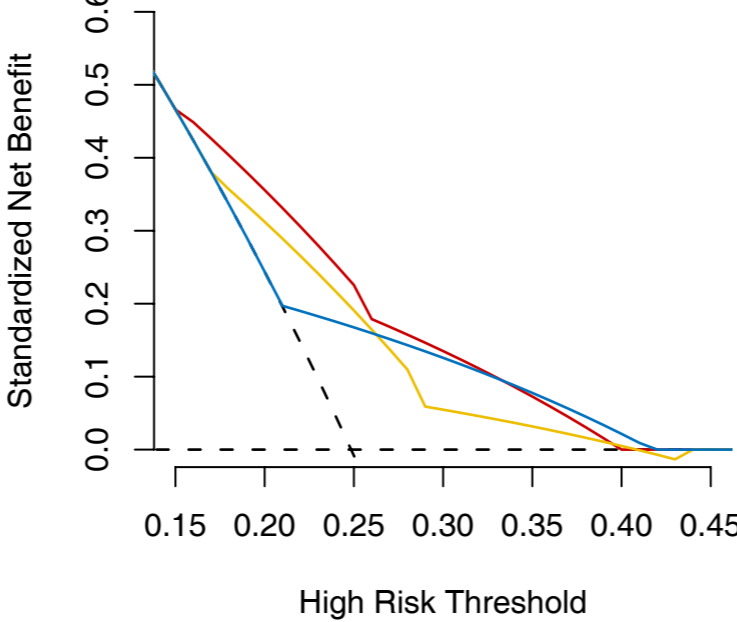

F: DCA-DMFS

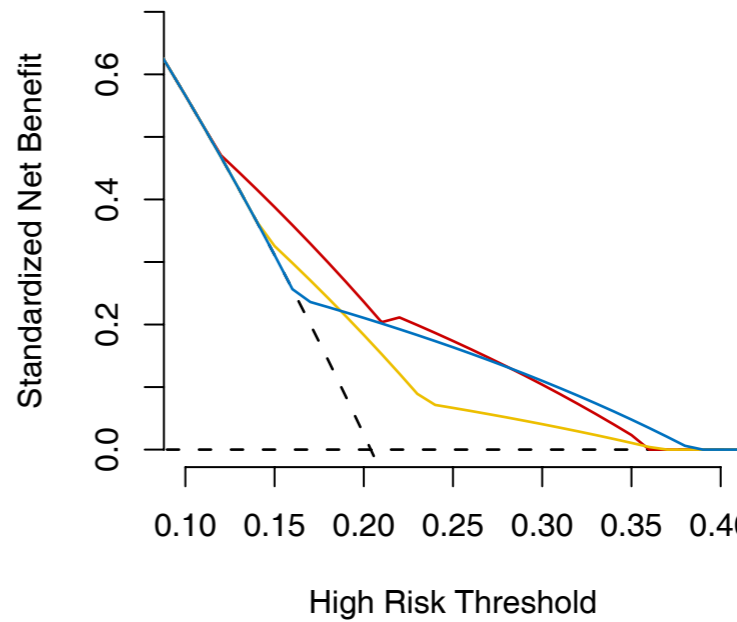

Supplement: Supplementary file 5 — Figure S5. [file CAM4-12-15079-s004.pdf]
