# Supplementary material for: Construction of refined staging classification systems integrating FIGO/T‐categories and corpus uterine invasion for non‐metastatic cervical cancer
Source: Cancer Med. 2023 Jun 16;12(14):15079–89. doi: 10.1002/cam4.6179 (PMC10417195; doi:10.1002/cam4.6179)
Supplement: Supplementary file 2 — Figure S2. [file CAM4-12-15079-s002.pdf]

A

		FIGO stage I	FIGO stage II	FIGO stage III	Stage I'	Stage II'	Stage III'
5-Y OS	Corpus Uteri Negative	90.8%	82.1%	69.5%	90.8%	82.1%	68.5%
	Corpus Uteri Positive	69.7%	63.7%				
5-Y PFS	Corpus Uteri Negative	86.6%	79.2%	61.5%	86.6%	76.2%	60.0%
	Corpus Uteri Positive	52.5%	57.5%				
5-Y DMFS	Corpus Uteri Negative	89.5%	82.9%	67.2%	89.5%	82.9%	64.9%
	Corpus Uteri Positive	56.8%	59.1%				

B

Hazard Ratio		FIGO stage I	FIGO stage II	FIGO stage III	Stage I'	Stage II'	Stage III'
OS	Corpus Uteri Negative	Ref.	2.10	4.10	Ref.	1.95	3.89
	Corpus Uteri Positive	3.22	3.77				
PFS	Corpus Uteri Negative	Ref.	1.93	3.29	Ref.	1.86	3.43
	Corpus Uteri Positive	3.17	3.14				
DMFS	Corpus Uteri Negative	Ref.	1.77	3.72	Ref.	1.72	3.85
	Corpus Uteri Positive	4.02	3.45				

C

		T1	T2	T3	T1'	T2'	T3'
5-Y OS	Corpus Uteri Negative	89.7%	78.8%	67.9%	89.7%	78.8%	68.0%
	Corpus Uteri Positive	69.2%	67.4%				
5-Y PFS	Corpus Uteri Negative	85.0%	72.8%	57.8%	85.0%	72.8%	59.0%
	Corpus Uteri Positive	57.2%	60.7%				
5-Y DMFS	Corpus Uteri Negative	88.0%	80.0%	61.5%	88.0%	80.0%	63.1%
	Corpus Uteri Positive	60.3%	65.5%				

D

Hazard Ratio		T1	T2	T3	T1'	T2'	T3'
OS	Corpus Uteri Negative	Ref.	2.10	4.10	Ref.	2.10	3.79
	Corpus Uteri Positive	3.22	3.77				
PFS	Corpus Uteri Negative	Ref.	1.93	3.29	Ref.	1.93	3.20
	Corpus Uteri Positive	3.17	3.14				
DMFS	Corpus Uteri Negative	Ref.	1.77	3.72	Ref.	1.77	3.66
	Corpus Uteri Positive	4.02	3.45				

90% 70% 50%
