# Supplementary material for: Construction of refined staging classification systems integrating FIGO/T‐categories and corpus uterine invasion for non‐metastatic cervical cancer
Source: Cancer Med. 2023 Jun 16;12(14):15079–89. doi: 10.1002/cam4.6179 (PMC10417195; doi:10.1002/cam4.6179)
Supplement: Supplementary file 6 — Figure S6. [file CAM4-12-15079-s006.pdf]

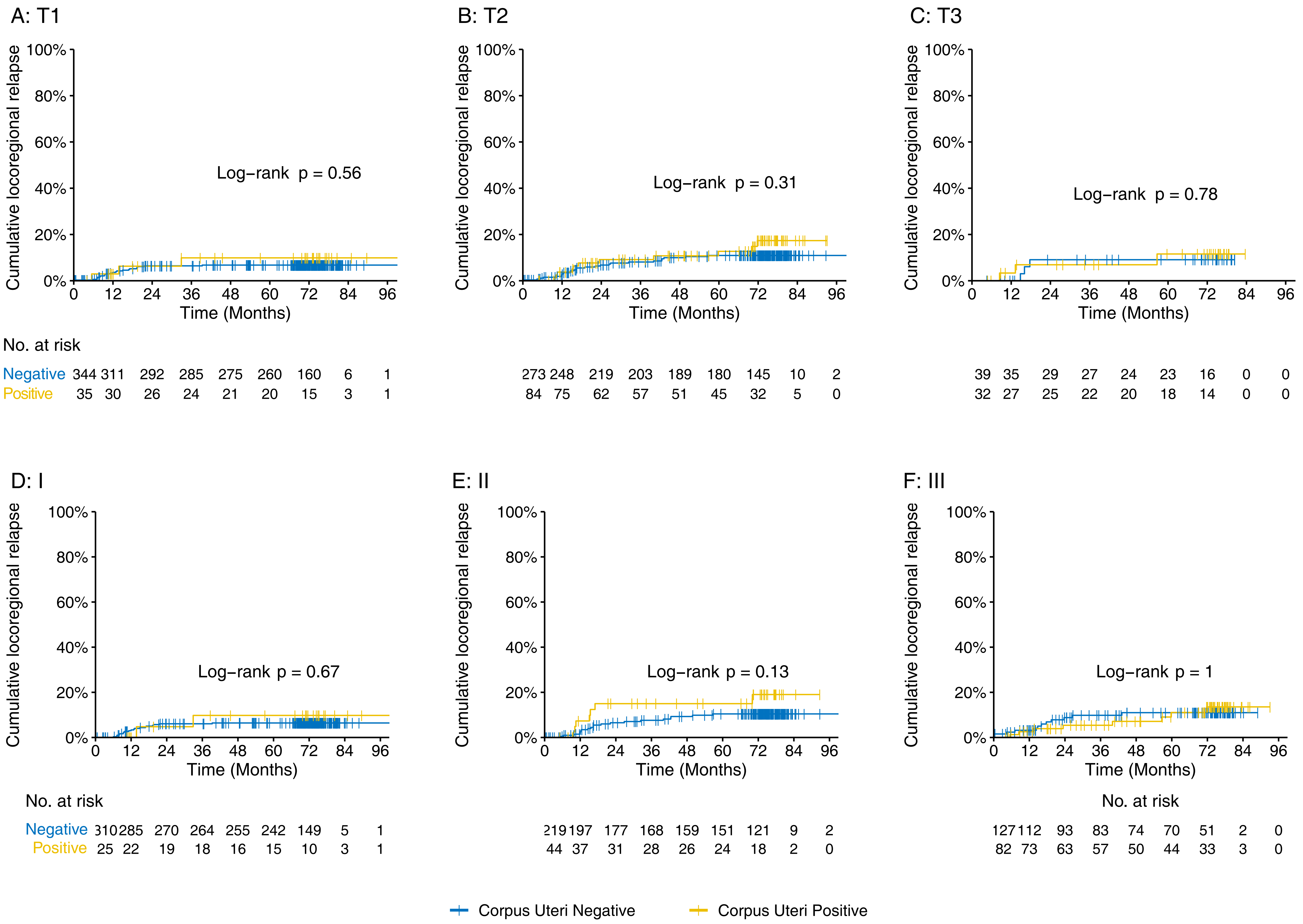
A: T1
B: T2
C: T3
D: I
E: II
F: III
Cumulative locoregional relapse
100%
80%
60%
40%
20%
0%
0 12 24 36 48 60 72 84 96
Time (Months)
Log−rank p = 0.56
Log−rank p = 0.31
Log−rank p = 0.78
Log−rank p = 0.67
Log−rank p = 0.13
Log−rank p = 1
No. at risk
Negative 344 311 292 285 275 260 160 6 1
Positive 35 30 26 24 21 20 15 3 1
273 248 219 203 189 180 145 10 2
84 75 62 57 51 45 32 5 0
39 35 29 27 24 23 16 0 0
32 27 25 22 20 18 14 0 0
Negative 310 285 270 264 255 242 149 5 1
Positive 25 22 19 18 16 15 10 3 1
219 197 177 168 159 151 121 9 2
44 37 31 28 26 24 18 2 0
127 112 93 83 74 70 51 2 0
82 73 63 57 50 44 33 3 0
Corpus Uteri Negative
Corpus Uteri Positive
