# Supplementary material for: Construction of refined staging classification systems integrating FIGO/T‐categories and corpus uterine invasion for non‐metastatic cervical cancer
Source: Cancer Med. 2023 Jun 16;12(14):15079–89. doi: 10.1002/cam4.6179 (PMC10417195; doi:10.1002/cam4.6179)
Supplement: Supplementary file 7 — Table S1. [file CAM4-12-15079-s005.docx]

**Appendix Table 1.** Comparison of clinical outcome segregated by Corpus Uteri Status.

|  | Corpus Uteri Negative | Corpus Uteri Positive |
| --- | --- | --- |
| **Overall survival** | |  |
| 5-Year rate  (95% CI) | 84.2%  (82.7%-85.7%) | 66.7%  (62.7%-70.7%) |
| *P*-value | <0.001 | |
| HR (95% CI) | 2.50 (1.81-3.46) | |
| **Progression-free survival** | |  |
| 5-Year rate  (95% CI) | 79.2%  (77.6%-80.8%) | 61.1%  (57.0%-65.2%) |
| *P*-value | <0.001 | |
| HR (95% CI) | 2.27 (1.69-3.06) | |
| **Distant metastasis-free survival** | |  |
| 5-Year rate  (95% CI) | 83.6%  (82.1%-85.1%) | 61.3%  (57.2%-65.4%) |
| *P*-value | <0.001 | |
| HR (95% CI) | 2.79 (2.02-3.84) | |

**Appendix Table 2.** Multivariate analyses of potential prognostic factors in clinical outcomes.

|  | **FIGO stage** | |  | **T stage** | |
| --- | --- | --- | --- | --- | --- |
|  | **HR (95%CI)** | ***P*** |  | **HR (95%CI)** | ***P*** |
| **Overall survival** |  |  |  |  |  |
| Age (≥50 *vs*. <50) |  | NS |  |  | NS |
| Histologic type (AC *vs.* SCC) | 2.27 (1.43-3.60) | <0.001 |  | 2.10 (1.34-3.30) | <0.001 |
| Histologic type (ASC *vs.* SCC) | 1.17 (0.42-3.20) | 0.767 |  | 1.03 (0.38-2.81) | 0.954 |
| Histologic type (Other *vs.* SCC) | 3.00 (1.54-5.84) | <0.001 |  | 2.76 (1.45-5.28) | 0.002 |
| Stage (II *vs*. I) | 1.98 (1.26-3.13) | 0.003 |  | 2.04 (1.41-2.96) | <0.001 |
| Stage (III *vs*. I) | 2.38 (1.45-3.91) | <0.001 |  | 3.09 (1.83-5.15) | <0.001 |
| Corpus Uteri (Positive *vs*. Negative) | 1.77 (1.25-2.53) | 0.002 |  | 1.91 (1.35-2.70) | <0.001 |
| Treatment (CRT *vs.* S+A) |  | NS |  |  | NS |
| Treatment (RT *vs.* S+A) |  | NS |  |  | NS |
| Treatment (S *vs.* S+A) |  | NS |  |  | NS |
| **Progression-free survival** |  |  |  |  |  |
| Age (≥50 *vs*. <50) |  | NS |  |  | NS |
| Histologic type (AC *vs.* SCC) | 2.08 (1.30-3.33) | 0.002 |  | 2.06 (1.28-3.30) | 0.003 |
| Histologic type (ASC *vs.* SCC) | 1.56 (0.68-3.60) | 0.296 |  | 1.56 (0.68-3.60) | 0.294 |
| Histologic type (Other *vs.* SCC) | 3.08 (1.67-5.68) | <0.001 |  | 3.10 (1.69-5.70) | <0.001 |
| Stage (II *vs*. I) | 1.73 (1.16-2.58) | 0.007 |  | 1.56 (1.08-2.24) | 0.017 |
| Stage (III *vs*. I) | 1.88 (1.20-2.94) | 0.006 |  | 1.50 (0.84-2.70) | 0.174 |
| Corpus Uteri (Positive *vs*. Negative) | 1.74 (1.25-2.42) | <0.001 |  | 1.74 (1.26-2.41) | <0.001 |
| Treatment (CRT *vs.* S+A) | 1.82 (1.25-2.65) | 0.002 |  | 1.90 (1.25-2.90) | 0.003 |
| Treatment (RT *vs.* S+A) | 0.99 (0.56-1.73) | 0.961 |  | 1.01 (0.57-1.79) | 0.980 |
| Treatment (S *vs.* S+A) | 1.15 (0.74-1.80) | 0.537 |  | 1.08 (0.69-1.68) | 0.734 |
| **Distant metastasis-free survival** |  |  |  |  |  |
| Age (≥50 *vs*. <50) |  | NS |  |  | NS |
| Histologic type (AC *vs.* SCC) | 2.37 (1.50-3.75) | <0.001 |  | 2.19 (1.39-3.45) | <0.001 |
| Histologic type (ASC *vs.* SCC) | 1.78 (0.77-4.12) | 0.178 |  | 1.80 (0.78-4.17) | 0.168 |
| Histologic type (Other *vs.* SCC) | 3.68 (2.00-6.80) | <0.001 |  | 3.82 (2.09-6.98) | <0.001 |
| Stage (II *vs*. I) | 1.56 (1.00-2.43) | 0.048 |  |  | NS |
| Stage (III *vs*. I) | 1.76 (1.09-2.85) | 0.022 |  |  | NS |
| Corpus Uteri (Positive *vs*. Negative) | 2.06 (1.45-2.93) | <0.001 |  | 2.30 (1.65-3.22) | <0.001 |
| Treatment (CRT *vs.* S+A) | 1.93 (1.27-2.94) | 0.002 |  | 2.29 (1.54-3.40) | <0.001 |
| Treatment (RT *vs.* S+A) | 1.09 (0.59-2.01) | 0.779 |  | 1.27 (0.70-2.31) | 0.430 |
| Treatment (S *vs.* S+A) | 0.84 (0.50-1.41) | 0.507 |  | 0.69 (0.42-1.14) | 0.146 |

Abbreviation: NS: not significant; SCC: Squamous Cell Carcinoma; AC: Adenocarcinoma; ASC: Adenosquamous Carcinoma; IQR: interquartile range; NA: not available; S: surgery; CRT: chemo-radiotherapy; RT: radiotherapy; A: adjuvant therapy.

**－:** Corpus Uteri status was not included in this analysis.

**Appendix Table 3.** General characteristics of patients stratified by RPA stage groupings.

|  | **Proposed RPA-FIGO Stage Groupings** | | | |  | **Proposed RPA-T Stage Groupings** | | | |
| --- | --- | --- | --- | --- | --- | --- | --- | --- | --- |
| Characteristic | I'  (n=310) | II'  (n=219) | III'  (n=280) | *P*-value |  | T1''  (n=345) | T2' (n=274) | T3' (n=190) | *P*-value |
| Age (years) | |  |  | <0.001 |  |  |  |  | <0.001 |
| Median  IQR | 46  38-51 | 54  46-60 | 51  45-57 |  |  | 46  39-51 | 54  46-59 | 52  46-58 |  |
| Smoking, *n* (%) | |  |  | 0.680 |  |  |  |  | 0.906 |
| None | 306  (98.7) | 218  (99.5) | 277  (98.9) |  |  | 341 (98.8) | 272 (99.3) | 188 (98.9) |  |
| Yes | 4 (1.3) | 1 (0.5) | 3 (1.1) |  |  | 4 (1.2) | 2 (0.7) | 2 (1.1) |  |
| Drinking, *n* (%) |  |  |  | 0.917 |  |  |  |  | 0.910 |
| None | 307  (99) | 216  (98.6) | 277  (98.9) |  |  | 342 (99.1) | 270 (98.5) | 188 (98.9) |  |
| Yes | 3 (1) | 3 (1.4) | 3 (1.1) |  |  | 3 (0.9) | 4 (1.5) | 2 (1.1) |  |
| WHO histologic type, *n* (%) | | |  | <0.001 |  |  |  |  | <0.001 |
| SCC | 237  (76.5) | 197  (90) | 240  (85.7) |  |  | 266 (77.1) | 245 (89.4) | 163 (85.8) |  |
| AC | 58 (18.7) | 11 (5) | 24 (8.6) |  |  | 64 (18.6) | 13 (4.7) | 16 (8.4) |  |
| ASC | 7 (2.3) | 5 (2.3) | 7 (2.5) |  |  | 7 (2) | 6 (2.2) | 6 (3.2) |  |
| Other | 8 (2.6) | 6 (2.7) | 9 (3.2) |  |  | 8 (2.3) | 10 (3.6) | 5 (2.6) |  |
| FIGO 2018, *n* (%) | |  |  | <0.001 |  |  |  |  | <0.001 |
| I | 310 (100) | 0 (0) | 25 (8.9) |  |  | 345 (100) | 0 (0) | 35 (18.4) |  |
| II | 0 (0) | 219 (100) | 44 (15.7) |  |  | 0 (0) | 274 (100) | 84 (44.2) |  |
| III | 0 (0) | 0 (0) | 211 (75.4) |  |  | 0 (0) | 0 (0) | 71 (37.4) |  |
| Treatment, *n* (%) | |  |  | <0.001 |  |  |  |  | <0.001 |
| CRT | 3 (1) | 30 (13.7) | 82 (29.3) |  |  | 4 (1.2) | 42 (15.3) | 69 (36.3) |  |
| RT | 3 (1) | 19 (8.7) | 35 (12.5) |  |  | 5 (1.4) | 24 (8.8) | 28 (14.7) |  |
| S | 132 (42.6) | 22 (10) | 12 (4.3) |  |  | 135 (39.1) | 24 (8.8) | 7 (3.7) |  |
| S+A | 172 (55.5) | 148 (67.6) | 151 (53.9) |  |  | 201 (58.3) | 184 (67.2) | 86 (45.3) |  |

Abbreviation: SCC: Squamous Cell Carcinoma; AC: Adenocarcinoma; ASC: Adenosquamous Carcinoma; IQR: interquartile range; NA: not available; S: surgery; CRT: chemo-radiotherapy; RT: radiotherapy; A: adjuvant therapy.

**Appendix Table 4.** Multivariate analyses of potential prognostic factors in clinical outcomes.

|  | **RPA-FIGO stage** | |  | **RPA-T stage** | |
| --- | --- | --- | --- | --- | --- |
|  | **HR (95%CI)** | ***P*** |  | **HR (95%CI)** | ***P*** |
| **Overall survival** |  |  |  |  |  |
| Age (≥50 *vs*. <50) |  | NS |  |  | NS |
| Histologic type (AC *vs.* SCC) | 2.73 (1.65-4.52) | <0.001 |  | 2.51 (1.53-4.11) | <0.001 |
| Histologic type (ASC *vs.* SCC) | 1.31 (0.47-3.62) | 0.603 |  | 1.10 (0.40-3.00) | 0.859 |
| Histologic type (Other *vs.* SCC) | 3.4 (1.73-6.68) | <0.001 |  | 3.32 (1.72-6.39) | <0.001 |
| Stage (II' *vs*. I') or T' (T2' *vs*. T1') | 2.20 (1.31-3.69) | 0.003 |  | 2.27 (1.48-3.50) | <0.001 |
| Stage (III' *vs*. I') or T' (T3' *vs*. T1') | 3.73 (2.28-6.11) | <0.001 |  | 3.96 (2.60-6.03) | <0.001 |
| Treatment (CRT *vs.* S+A) | 1.71 (1.13-2.57) | 0.011 |  |  | NS |
| Treatment (RT *vs.* S+A) | 1.14 (0.64-2.04) | 0.664 |  |  | NS |
| Treatment (S *vs.* S+A) | 1.19 (0.70-2.01) | 0.528 |  |  | NS |
| **Progression-free survival** |  |  |  |  |  |
| Age (≥50 *vs*. <50) |  | NS |  |  | NS |
| Histologic type (AC *vs.* SCC) | 2.16 (1.34-3.47) | <0.001 |  | 2.22 (1.38-3.57) | <0.001 |
| Histologic type (ASC *vs.* SCC) | 1.59 (0.69-3.66) | 0.275 |  | 1.57 (0.68-3.62) | 0.287 |
| Histologic type (Other *vs.* SCC) | 3.09 (1.68-5.68) | <0.001 |  | 3.26 (1.78-5.96) | <0.001 |
| Stage (II' *vs*. I') or T' (T2' *vs*. T1') | 2.01 (1.28-3.15) | 0.003 |  | 1.94 (1.30-2.90) | <0.001 |
| Stage (III' *vs*. I') or T' (T3' *vs*. T1') | 3.32 (2.15-5.12) | <0.001 |  | 2.85 (1.85-4.39) | <0.001 |
| Treatment (CRT *vs.* S+A) | 1.73 (1.20-2.49) | 0.003 |  | 1.70 (1.17-2.48) | 0.006 |
| Treatment (RT *vs.* S+A) | 0.98 (0.56-1.71) | 0.944 |  | 1.01 (0.58-1.76) | 0.979 |
| Treatment (S *vs.* S+A) | 1.31 (0.83-2.06) | 0.252 |  | 1.16 (0.74-1.81) | 0.515 |
| **Distant metastasis-free survival** |  |  |  |  |  |
| Age (≥50 *vs*. <50) |  | NS |  |  | NS |
| Histologic type (AC *vs.* SCC) | 2.51 (1.58-3.99) | <0.001 |  | 2.49 (1.56-3.96) | <0.001 |
| Histologic type (ASC *vs.* SCC) | 1.89 (0.82-4.35) | 0.135 |  | 1.79 (0.78-4.14) | 0.172 |
| Histologic type (Other *vs.* SCC) | 3.72 (2.03-6.82) | <0.001 |  | 3.94 (2.16-7.21) | <0.001 |
| Stage (II' *vs*. I') or T' (T2' *vs*. T1') | 1.91 (1.14-3.20) | 0.014 |  | 1.81 (1.15-2.85) | 0.010 |
| Stage (III' *vs*. I') or T' (T3' *vs*. T1') | 3.62 (2.26-5.81) | <0.001 |  | 3.21 (2.03-5.09) | <0.001 |
| Treatment (CRT *vs.* S+A) | 1.79 (1.20-2.69) | 0.005 |  | 1.74 (1.15-2.64) | 0.009 |
| Treatment (RT *vs.* S+A) | 1.08 (0.59-1.96) | 0.802 |  | 1.11 (0.61-2.02) | 0.742 |
| Treatment (S *vs.* S+A) | 0.98 (0.57-1.67) | 0.934 |  | 0.85 (0.50-1.43) | 0.531 |

Abbreviation: NS: not significant; SCC: Squamous Cell Carcinoma; AC: Adenocarcinoma; ASC: Adenosquamous Carcinoma; IQR: interquartile range; SCCA: Squamous cell carcinoma antigen; NA: not available; S: surgery; CRT: chemo-radiotherapy; RT: radiotherapy; A: adjuvant therapy.

**Appendix Table 5.** Distribution of patients in the RPA groupings, compared to the 2018 FIGO and 9^th^ edition T classification systems in the cohort.

|  | **AJCC Stage** | **N** | **Proposed RPA staging** |
| --- | --- | --- | --- |
| **FIGO** | Stage I | 335 (41.4%) | Stage I': 310 (92.5%) |
|  |  |  | Stage III': 25 (7.5%) |
|  | Stage II | 263 (32.5%) | Stage II': 219 (83.3%) |
|  |  |  | Stage III': 44 (16.7%) |
|  | Stage III | 211 (26.1%) | Stage III': 211 (100%) |
| **T-category** | T1 | 380 (47.0%) | T1': 345 (90.8%) |
|  |  |  | T3': 35 (9.2%) |
|  | T2 | 358 (44.2%) | T2': 274 (76.5%) |
|  |  |  | T3': 84 (23.5%) |
|  | T3 | 71 (8.8%) | T3': 71 (100%) |

**Appendix Table 6.** Comparison of failure patterns of patients segregated by Corpus Uteri Status.

|  | Corpus Uteri  Negative | Corpus Uteri  Positive | *P*-value |
| --- | --- | --- | --- |
| Distant metastasis |  |  | **<0.001** |
| No | 596 (90.6%) | 111 (73.5%) |  |
| Yes | 62 (9.4%) | 40 (26.5%) |  |
| Loco-regional relapse | |  | 0.161 |
| No | 607 (92.2%) | 134 (88.7%) |  |
| Yes | 51 (7.8%) | 17 (11.3%) |  |

**Figure legends**

**Appendix Figure 1. Comparison of survival outcomes among patients with or without corpus uteri invasion.**

**Appendix Figure 2. Development of refined staging systems for cervical cancer incorporating corpus uteri status.** Survival rates of different patients respectively with FIGO stage I-II (or T1-2) incorporating corpus uteri status, FIGO stage III(or T3) and the proposed FIGO stage I'-III' (or T1'-3' )(A,C), and the corresponding hazard ratios for 5-year overall survival (OS), 5-year progression-free survival (PFS) and distant metastasis-free survival (DMFS)(B,D).

**Appendix Figure 3. Kaplan-Meier curves for progression-free survival and distant metastasis–free survival stratified by the RPA-FIGO risk classifications (A-B) or the RPA-T risk classifications (C-D).**

**Appendix Figure 4. Receiver operating characteristic curve (A-C) and decision curve analysis (D-F) comparing the efficacy of survival prediction between the proposed RPA-FIGO staging system and the 2018 FIGO classification.**

**Appendix Figure 5. Receiver operating characteristic curve (A-C) and decision curve analysis (D-F) comparing the efficacy of survival prediction between the proposed RPA-T staging system and the 9^th^ TNM stage schema.**

**Appendix Figure 6. Cumulative locoregional relapse stratified by the corpus uteri status within each T category (A-C) and FIGO stage (D-F).**
